# Reloading Pupils’ Batteries: Impact of Green Spaces on Cognition and Wellbeing

**DOI:** 10.3390/ijerph15061205

**Published:** 2018-06-08

**Authors:** Peter Wallner, Michael Kundi, Arne Arnberger, Renate Eder, Brigitte Allex, Lisbeth Weitensfelder, Hans-Peter Hutter

**Affiliations:** 1 Department of Environmental Health, Center for Public Health, Medical University Vienna, Kinderspitalgasse 15, 1090 Vienna, Austria; peter.wallner4@gmail.com (P.W.); michael.kundi@meduniwien.ac.at (M.K.); lisbeth.weitensfelder@meduniwien.ac.at (L.W.); 2 Institute of Landscape Development, Recreation and Conservation Planning, Department of Landscape, Spatial and Infrastructure Sciences, University of Natural Resources and Life Sciences, Peter-Jordan-Straße 82/I, 1190 Vienna, Austria; arne.arnberger@boku.ac.at (A.A.); renate.eder@boku.ac.at (R.E.); brigitte.allex@boku.ac.at (B.A.)

**Keywords:** green spaces, park, forest, pupils, adolescents, school, break, cognition, d2 test, wellbeing

## Abstract

Cognitive functioning and academic performance of pupils depend on regular breaks from classroom work. However, it is unclear which settings during such breaks provide the best environment to restore cognitive performance and promote wellbeing of adolescent pupils. Therefore, we investigated the effects of staying in different urban green spaces during breaks. Sixty-four pupils (16–18 years old) participated in a cross-over experiment. They were placed into one of three settings (small park, larger park, forest) for one hour during a lunch break. Wellbeing was assessed four times (Nitsch scale), and a cognitive test (d2-R Test of Attention) was applied in the classrooms before and after the break. Wellbeing was almost always highest after the stay in the green spaces. However, a sustained effect was only found for the forest. Concentration performance values of the d2-R test were significantly higher after the pupils’ stay in green spaces for all sites. The highest increase of performance was found for the larger park type. In conclusion, this pilot study showed that study breaks in green spaces improved wellbeing and cognitive performance of adolescents. It also found that larger green spaces, either parks or forests, have stronger positive impacts on wellbeing and cognitive performance than small parks.

## 1. Introduction

Long school days challenge the wellbeing and cognitive performance of pupils. Time to recover is crucial, as cognitive functioning and academic performance depend on regular breaks from classroom work [1]. This applies for both children and adolescents [1,2]. However, it is unclear which settings (for example, green spaces) during such breaks provide the optimal environment to prevent deterioration in performance and wellbeing of adolescent pupils.

To explain the effects of nature on cognition and wellbeing, two complementary theories are primarily applied. These are the Attention Restoration Theory [3] and the Stress Reduction Theory [4]. Empirical evidence agrees with both theories [5] (i.e., that natural environments are more restorative than urban ones), but the theories are based on different backgrounds: Attention Restoration Theory suggests that people are driven to a restorative place by mental fatigue, while the drive in Stress Reduction Theory is physiological stress [5]. Though it might be possible that attention restoration occurs via stress reduction, a study investigating a mediation effect between the two processes found them to be distinct [6]. Attention Restoration Theory proposes that contact with nature can restore people’s directed attention [3,7,8]. Fascination plays an important role for attention restoration [9], with fascinating environments being interesting without requiring directed attention. A recent systematic review states that although the evidence on the association between green spaces and cognition is inadequate, “it is suggestive for beneficial associations between such an exposure and cognitive development in childhood and cognitive function in adulthood” [10].

With regard to adolescents, a study of students’ performance in 101 public high schools in Michigan revealed a positive association between nature exposure (at school) and performance [11]. Most studies, however, focus on already grown up students: university dormitory residents (72 undergraduate students) scored better in tests of directed attention if their windows showed more natural views [12]. After a 50- to 55-min walk in an arboretum, the cognitive performance (backwards digit-span test) of 38 students (mean age 22.6 years) improved significantly [13]. Positive effects on cognitive performance can also be found after short exposure times: the results of the backwards digit-span test even improved after 12 students had viewed pictures of nature for approximately 10 min [13]. In another study, viewing a green/flowered vs. concrete rooftop for just 40 s improved attention control of university students [14]. Such results support the Attention Restoration Theory with performance results. Other studies support the theory with results from subjective ratings: when college students imagined themselves to be cognitively fatigued, they rated settings that had no views of real or simulated nature (murals) to be least restorative [15].

Stress Reduction Theory [4], which assumes that natural environments can reduce stress, is empirically supported. Studies have confirmed that exposure to natural settings can lead to increased wellbeing and stress reduction (e.g., [4,16,17,18,19,20,21,22]). There is evidence for a causal relationship between surrounding greenness and mental health in adults, but results for children are inadequate [23]. One study found that for adolescents, the surrounding greenness around a residence (1250 m-buffer) is associated with 11% lower odds of high depressive symptoms [24], but results of single studies have to be interpreted carefully due to the high dependency on the measure type and influences of moderator variables: an approach testing several pathways of how green space and youth mental health are connected resulted in different conclusions, depending on the method used [25].

Although adolescents also value nature [26], there is some evidence that preference for natural areas among adolescents is lower than in other age groups [27,28]. Recalled feelings of restoration (such as feeling relaxed and refreshed) after visits to natural environments were lowest in the age group of 16–24 years [29], and one study even found nature to be “scary, disgusting and uncomfortable” from an adolescent’s point of view [30]. A positive and stress-reducing effect of nature on adolescents might also differ depending on whether nature is away from home or not [31], with positive effects only for sites being away.

Chawla et al. [32] found that green schoolyards can reduce stress in children and adolescents. A 15-min springtime walk in an urban park led to lower sympathetic nervous activity and an improved mood state in young Japanese males (mean age 21.2 years) [33]. In adolescent girls from Finland, nature evoked mainly positive feelings [34].

Taken together, there is research on the positive effects of exposure to green spaces for both complementary theories, but data on the effects of study breaks spent in green spaces and nature on adolescents’ cognitive performance and wellbeing is sparse. In addition, little is known about the effects of specific landscape types on wellbeing and cognitive performance [35,36,37,38], in particular for adolescents. Also, results about different green sites are contradictious: a laboratory experiment with university students found a stronger recovery from emotional stress for urban green sites compared to built urban sites, but no significant differences between the different green sites [39]. Contrary to that, another study that showed pictures to students concluded that perceived restoration increased with the level of naturalness [36], though in the latter study some questionnaire items might have led to a bias (e.g., asking how much potential for exploration the site offers). Nevertheless, further investigation is required in regard to whether different levels of greenery lead to different levels of restoration. Therefore, within a multidisciplinary approach this study investigated the effects of staying in different urban green spaces during breaks on the wellbeing and cognitive performance of adolescents. We hypothesized that short-term stays in green spaces with different degrees of naturalness have different effects on wellbeing (hypothesis 1) and cognitive performance (hypothesis 2).

## 2. Materials and Methods

Healthy pupils (*n* = 64; 16–18 years old) from three schools in Vienna participated in a cross-over field experiment as a class. Every class was placed into three different settings (inner urban small and heavily used park with a few trees and surrounded by heavily used streets and dense residential areas, a larger park with some tree clumps, or a larger broadleaved forest with some scattered meadows and low visitor numbers) for one hour during a lunch break after a challenging half-day at school (all students had five to six classes before the break, including 3 to 4 h of basic subjects). A minimum of a seven-day break was implemented between different exposures. The sequence of exposures was balanced across sites, and at each site, the same procedure was applied. Lunch was provided by the project team. After lunch, pupils took a walk together, followed by a brief relaxation period. The visits to the outdoor locations took place in May and June on days when it did not rain. Break sites were located approximately 20 min from school sites.

Wellbeing was assessed four times (prior to leaving school, on arrival at the allocated site, prior to leaving the green space, and at school after completing a cognitive test) to check for the momentary mood state. For assessment, the self-condition scale by Nitsch [40] was used. Participants characterized their actual state on 27 attributes (6-category scale from “does not apply at all” to “applies fully”) that map motivation and strain. The items belong to six dimensions: readiness for action, readiness for exertion, alertness, state of mood, tension/relaxation, and recuperation.

Cognitive performance was tested with a d2-R test [41], a timed test of selective attention [42]. For this test, the task required the subjects to cross out all letter “d’s” that have two dashes. The cognitive test was applied in the classrooms before and after the break.

### Statistical Analyses

In total, data from 60 students (30 male, 30 female) were available (age: 16.6 ± 0.6). Scores of the self-condition scale were transformed into stanine values and the difference to the baseline value for each pupil was evaluated by analysis of variance. Since all pupils from one school had the same sequence of site visits, pupils were nested within the school factor. The other factors were the within-subject factors time point (arrival at the site, before leaving the site, and back in the class room) and site (small urban park, large urban park, forest). Differences between the forest site from the two other sites were evaluated by linear contrasts for each time point. Residuals were tested for normality by Kolmogorov-Smirnov tests with Lilliefors corrected *p*-values. Sphericity was tested by Mauchly’s test and symmetry of the covariance matrix by Box M tests. Raw values of the d2-R test (total performance score) were transformed into standard T values according to the age-specific norms. Differences after-before stay at the site were evaluated by analysis of variance analogous to the self-condition scale except for the time factor. All analyses were performed by Statistica 10.0 (StatSoft, Tulsa, OK, USA).

## 3. Results

### 3.1. Wellbeing

**Figure 1:**
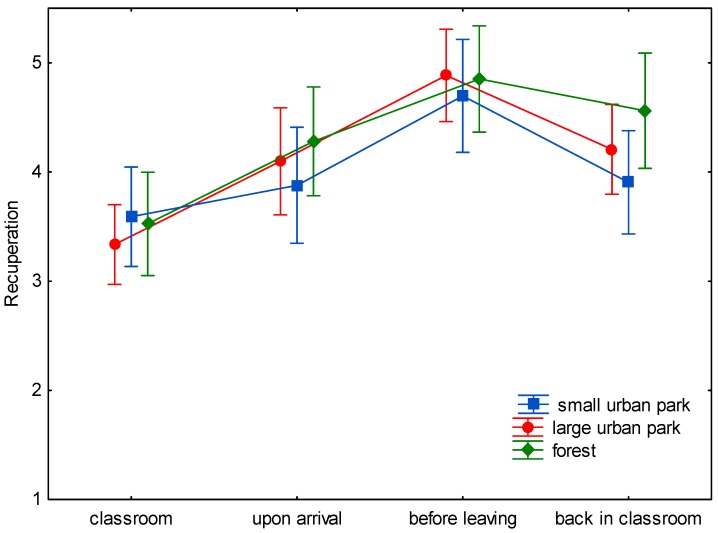
Mean and 95% confidence intervals of the stanine values of recuperation (Nitsch scale) at the four time points and three locations.

**Figure 2:**
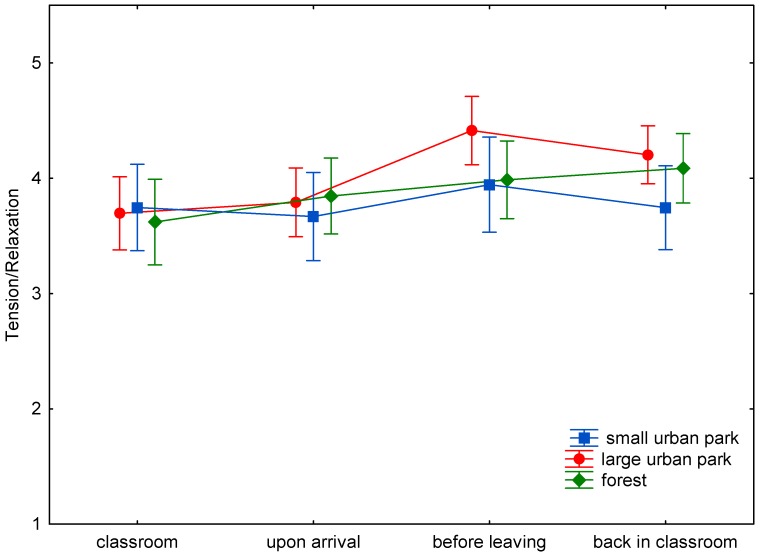
Mean and 95% confidence intervals of the stanine values of tension/relaxation (Nitsch scale) at the four time points and three locations.

**Figure 3:**
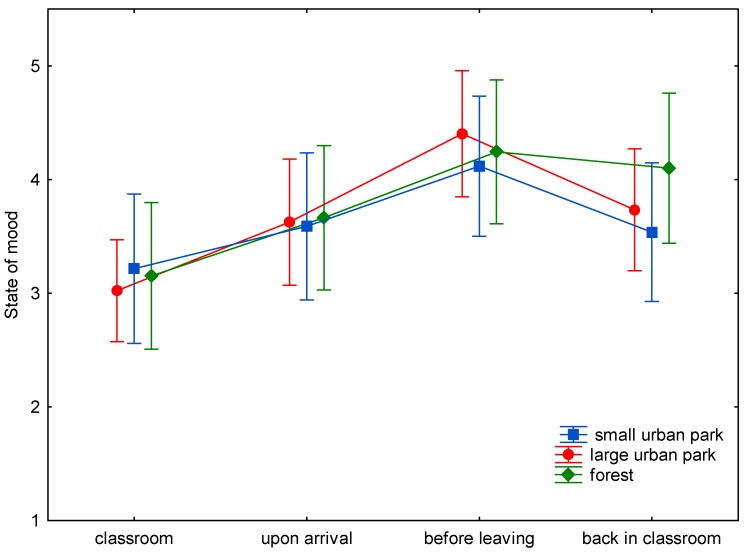
Mean and 95% confidence intervals of the stanine values of state of mood (Nitsch scale) at the four time points and three locations.

**Figure 4:**
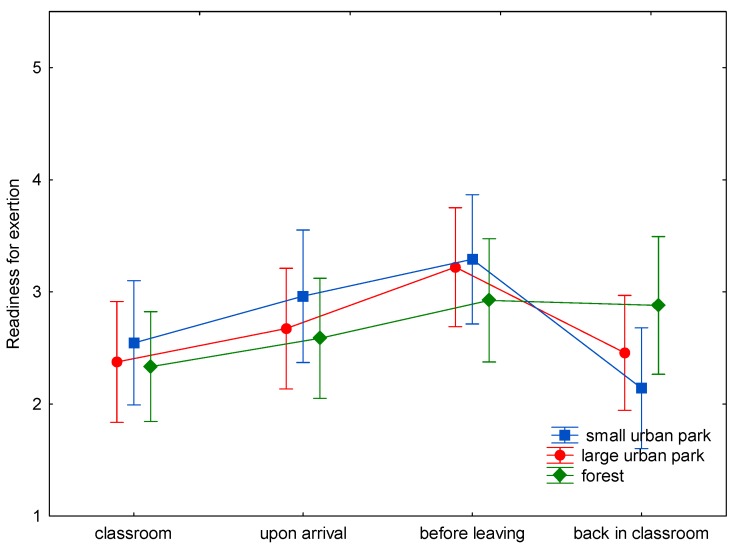
Mean and 95% confidence intervals of the stanine values of readiness for exertion (Nitsch scale) at the four time points and three locations.

**Figure 5:**
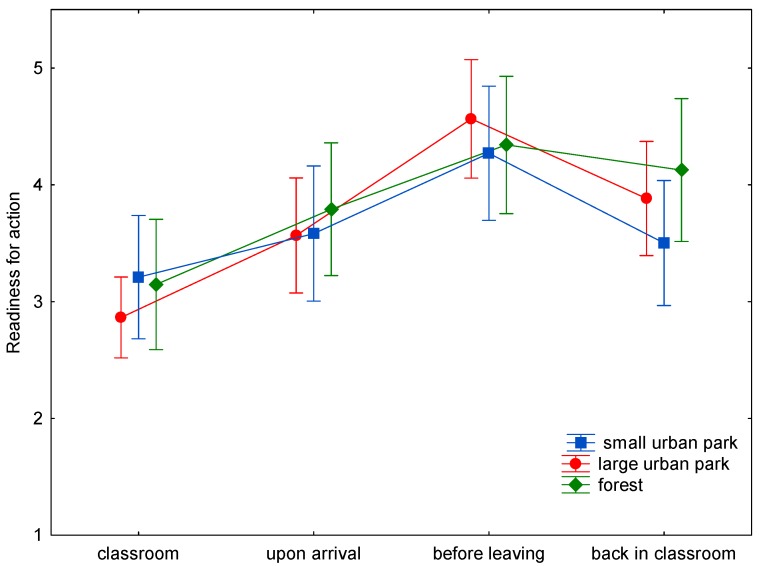
Mean and 95% confidence intervals of the stanine values of readiness for action (Nitsch scale) at the four time points and three locations.

**Figure 6:**
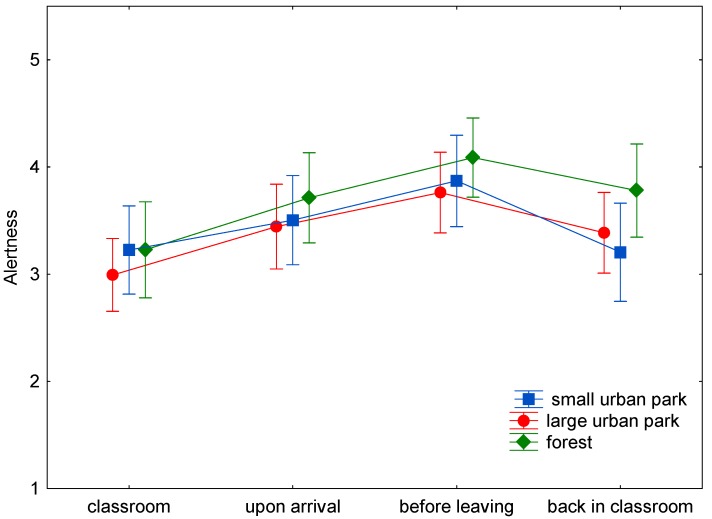
Mean and 95% confidence intervals of the stanine values of alertness (Nitsch scale) at the four time points and three locations.

The baseline assessment of wellbeing demonstrated only nonsignificant differences between the three study sites (Figure 1, Figure 2, Figure 3, Figure 4, Figure 5 and Figure 6). This was true for all six dimensions of the self-condition scale (recuperation, tension/relaxation, state of mood, readiness for action, readiness for exertion, and alertness). There were significant differences (*p* < 0.05) between time points in all dimensions of the scale. The condition of the pupils showed a trend to improve shortly after arrival in the respective green space and mostly reached a significant improvement after a longer stay, namely before leaving the site. With one exception, wellbeing was always highest before leaving the green spaces.

Statistically significant differences (and in one case a trend towards significance) between the three green space types were found for the dimensions recuperation, tension/relaxation, state of mood, readiness for action, and readiness for exertion. For the forest type, the decrease of wellbeing after return into the classroom was significantly less expressed than after the stay in the small or large urban park (readiness for action: *p* < 0.001; readiness for exertion: *p* = 0.027; state of mood: *p* < 0.001; tension/relaxation: *p* < 0.001) and a trend was found for recuperation (*p* = 0.089).

While recuperation values declined upon return into the classroom by 0.82 and 0.66 stanine units after the stay in the small and large urban park, respectively, the decline tended to be less expressed (0.32 stanine units) after return from the forest. 

Ratings of relaxation increased by 0.13 stanine units on average back in the classroom after stays in the forest, which was statistically different (*p* < 0.001) from the declines observed upon return from the small and large urban parks (decline of 0.21 stanine units in both cases).

State of mood declined in the classroom on average by 0.57 stanine units after stays in the small urban park, and by 0.67 units after stays in the large urban park, while this decline was much less expressed after stays in the forest (0.14 stanine units, *p* < 0.001).

A highly significant, less pronounced decline after returning from the forest was also found for readiness for exertion. After returning from the small and large urban parks, readiness for exertion scores declined by 1.19 and 0.78 stanine units, respectively, while after stays in the forest the decline amounted to 0.09 units only.

Similarly, for readiness for action, the decline was more pronounced after returning from small and large urban parks (0.46 and 0.59 stanine units, respectively) than after returning from the forest (decline of 0.05 stanine units).

No significant differences between the sites were obtained for alertness. Values declined by 0.64, 0.35, and 0.28 stanine units after stays in the small or large urban park and the forest, respectively. 

### 3.2. Cognitive Performance

**Figure 7:**
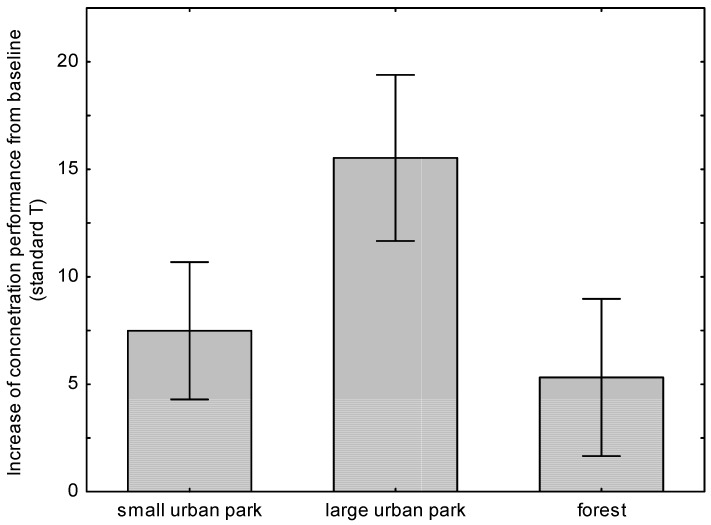
Mean differences after-before and 95% confidence intervals of concentration performance after three outdoor settings.

Concentration performance values of the d2-R test were significantly higher after the pupils’ stay in green spaces for all sites (*p* < 0.001). The increase after returning from the small urban park was 7.5 (standard deviation (SD) 9.7), after the large urban park was 15.5 (SD 11.7), and after returning from the forest was 5.3 (SD 11.0). The highest increase of performance was found for the larger park type (Figure 7), with this increase also being significantly higher (*p* = 0.008) than the increase after stays in the other green spaces.

## 4. Discussion

Few studies exist that assess the relationship between green spaces and the mood state of young people, especially in educational settings. Li and Sullivan [6], for example, conducted an experiment with 94 high school students in which they investigated the effect of breaks in the classrooms with and without views to green landscapes. Such views led to significantly better stress recovery and attentional performance. A study in rural Austria investigated the effects of greening a schoolyard on middle school pupils. The renovation resulted in reduced stress levels and enhanced wellbeing [43]. In elementary school children, vegetation volume of school playgrounds was associated with perceived restorativeness [44]. Green schoolyards can be seen as “havens from stress” [32].

In a study on young Japanese males, significant differences between a short springtime walk in an urban park and a walk in a nearby city area were found [33]. Walking in the park had positive effects on the cardiovascular system (for example, reduced pulse rate) and mood and feelings (i.e., lower tension and anxiety) [33]. Similar effects were identified for walks in a park in winter [45].

In the present study (with a more complex design: three study sites, wellbeing was assessed four times on every site), time in the green spaces led to either a trend to improve (e.g., readiness for exertion) or a significant improvement (e.g., readiness for action) in wellbeing after spending some time in the green space. The improvement might be either due to the study break itself, the move to the study site, the green environment, or a combination of possible causes. Given the background of previous studies, it is probable that the green site accounts at least for part of the improvement. The condition of the pupils tended to improve shortly after arrival in the respective green space. At the end of the stay, scores were highest, with one exception: for tension/relaxation the scores were highest after the stay in the forest and return to the classroom. The wellbeing scores seem comparable for all green sites for the first measurement times (Figure 1, Figure 2, Figure 3, Figure 4, Figure 5 and Figure 6; overlapping confidence intervals especially for times before and upon arrival), but towards the end, the green site with the highest degree of naturalness (forest) shows a different trend: data shows that only the forest provided/evoked sustained effects in some wellbeing variables, measured after the pupils had completed the concentration test in the classroom. Scores were particularly high in readiness for exertion, mood, and tension/relaxation. The findings regarding wellbeing at least partly confirm the Stress Reduction Theory, even if effects did not sustain for all sites. Therefore, hypothesis 1 can partly be confirmed: while there are no significant differences between green spaces types during exposure, at least in some respects there are differences directly afterwards, with forests showing a more beneficial effect. Further investigation is required in order to determine why the effect of smaller green spaces did not seem to be sustainable. 

Regarding effects on cognitive performance, the participants’ concentration performances were significantly higher after their stays in all types of green spaces. The improvement was in accordance with a study on 38 students who took a 50- to 55-min walk in an arboretum. After the walk in nature, their cognitive performance (backwards digit-span) improved significantly [13]. With improvements of 5–15 standard *t* values, effects were rather big, though there is also a general improvement in concentration scores when the test is administered several times [46], diminishing the seemingly huge effect. Nevertheless, performance significantly differs throughout different green space sites, confirming hypothesis 2: 

Performance values of the d2-R test were significantly highest after the stay in the larger park, followed by the urban park and the large forest. An explanation might be that after the stay in the forest and the feeling of “being away” [7] the pupils were not motivated or attuned to perform a monotonous task. Another explanation could be that the semi-open landscape park, which provided a higher degree of mystery and fewer disturbance factors than the inner urban park and higher visual accessibility compared to the dense forest, had even more positive impacts on attention [3]. Finally, it could also be possible that the relatively simple monotonous task did not represent the best-fitting predictor to assess recovery from attentional fatigue: while results from previous studies are consistent regarding the impaired performance in divided attention caused by acute stress, they are inconsistent when it comes to selective attention [47], and the d2-R requires especially selective attention. Whatever the reason for the differences between sites, an improvement in concentration test results could be seen for all green spaces, which supports the Attention Restoration Theory. Nevertheless, it cannot be resolved completely why the forest showed less improvement than the large park.

Taken together, the results support both the Stress Reduction Theory and the Attention Restoration Theory and are in line with a previous study [6], but it has to be noted that the results were confounded by the method used [25]: stress reduction was assessed via self-rating questions, and it could be possible that other forms of assessment (e.g., physiological ones) might have showed different results. 

There are several other limitations to the given results: as we compared three types of green spaces, no conclusions can be drawn on the effect of other environmental sites (e.g., blue spaces, built environment). Also, all groups had a study break, and it is impossible to separate the effects of the break itself and the effect of the green spaces. Future studies could examine whether pupils benefit from short breaks in green environments or longer breaks in built environments. Another limitation can be seen in the study population: the sample consisted of pupils that had already finished compulsory education, therefore adolescents who drop out after the minimum amount of school years are not represented in the sample. Future research on whether pupils from different socioeconomic backgrounds might benefit from breaks in green sites should be encouraged.

Taken together, this study showed that study breaks in green spaces, especially larger ones, improved the wellbeing and cognitive performance of adolescents.

## 5. Conclusions

In conclusion, this pilot study found that larger green spaces, either parks or forests, have stronger positive impacts on wellbeing and cognitive performance than inner urban settings. Therefore, we recommend green schoolyards and easy accessible and larger green spaces with higher degrees of naturalness in the neighborhood of schools.
